# Diabetes and Mexicans: Why the Two Are Linked^1^

**Published:** 2004-12-15

**Authors:** Reynaldo Martorell

**Affiliations:** Department of Global Health, The Rollins School of Public Health of Emory University

## The Past

Obesity and diabetes were probably rare before the advent of agriculture. Our ancestors, hunters and gatherers for millennia, had varied but unpredictable diets. Studies of hunter-gatherers of the 20^th^ century suggest that animal sources dominated our ancient food basket, with plants (fruits, vegetables, and nuts) providing only 20% to 40% of total energy (1). Modern and presumably ancient hunter-gatherer populations, despite a high-fat, high-protein diet, were free of the signs and symptoms of noncommunicable diseases — a paradox. Perhaps energy needs were not always met, thus keeping body sizes in check; also, the relative lack of salt and simple carbohydrates, a mix of saturated and good fats, plenty of fiber, abundant micronutrients, a vigorous and active life, and less stress than we now endure may explain this finding. With the food supply uncertain, one would expect individuals with "thrifty" genotypes — genotypes that increase the ability to turn food to fat — to have a survival edge.

Agriculture brought a more predictable food supply but less variety. Crops failed from time to time, bringing on famines when stores of grain were depleted, but over time, agriculture allowed for increasingly larger populations, with thrifty genotypes thriving as before. Super foods — such as corn in Mesoamerica, the substance from which the Mayan gods in their fourth attempt were finally able to make man, according to the Popul Vuh, the sacred book of the Maya — came to provide as much as 80% or more of energy needs. Crowding brought new types of infections, which along with limited diets gave rise to the nutritional deficiencies that have plagued humankind in recent millennia. Agriculture fostered the development of highly stratified societies, and it became possible for a few to lead a life of luxury. Until the 20^th^ century, fatness was a marker of wealth.

## The Present

Most of my professional career has been devoted to the study of hunger and malnutrition in developing countries. As rates of child malnutrition decline in Latin America and in other developing countries, the prevalence of obesity is increasing rapidly, and I, like many of my colleagues, have begun to study both ends of the spectrum — namely, deficiency and excess (2).

**Figure 1:**
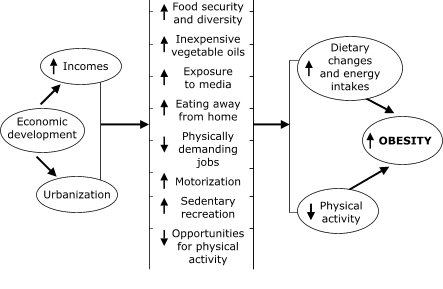
Possible causes of the nutrition transition and the emergence of obesity in developing countries. Adapted from Martorell and Stein, 2001 (2), and Popkin, 1994 (3).

Economic development and urbanization are the engines of the "nutrition transition" (3). Pathways include increased food security, the availability of cheap sources of fat in the form of vegetable oils, more eating away from home, the less arduous nature of modern jobs, and increases in sedentary recreation (notably television). These pathways have transformed dietary and physical activity patterns and, as a result, tipped the balance in favor of obesity (Figure 1).

Some populations may be more susceptible to obesity (e.g., Pacific Islanders, Native Americans) because of thrifty genotypes, as proposed by the geneticist Neel some years ago (4). Thrifty phenotypes may also increase susceptibility to obesity; some evidence suggests that poor intrauterine and infant nutrition may also "program" individuals to be metabolically thrifty, and if later times bring a life of abundance, these individuals will be at risk for developing chronic diseases such as diabetes (5).

## The "Supersizing" of the Mexican People

**Figure 2:**
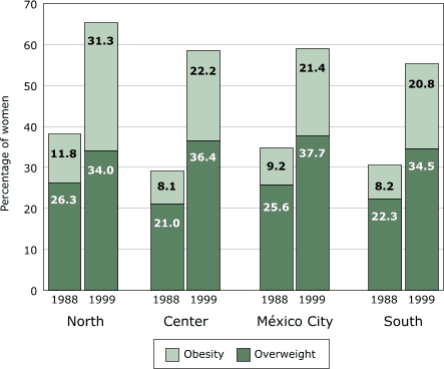
Levels of overweight (BMI = 25.0–29.9) and obesity (BMI ≥30.0) in 1988 and 1999 in women aged 18 to 49 in Mexico, by region. BMI indicates Body Mass Index. Data from Rivera et al, 2001 (6).

Mexico is a country far along the nutrition transition. The Mexican National Nutrition Survey 1999 showed that obesity (Body Mass Index [BMI] ≥30) among women aged 18 to 49 increased from 9% in 1988 to 24% in 1999 (6). If we add overweight (BMI = 25.0–29.9) to the mix, the percentage of overweight or obese women increased from 33% to 59% in just one decade. The 1999 survey also showed that the prevalence of stunting (low height-for-age, indicative of child undernutrition) among preschool children in the indigenous rural south of Mexico was 42%, as high as in many sub-Saharan African countries. Yet the problem of obesity grew alarmingly among all sectors of society. All socioeconomic groups, rural as well as urban areas, and all regions of Mexico, including the impoverished South, showed equally dramatic increases (Figure 2). Obesity and chronic diseases in Mexico can no longer be dismissed as problems of the rich. However, poor Mexicans have a double burden: child undernutrition in addition to obesity. As the nutrition transition unfolds even further, as it has in Chile, obesity becomes more common among the poor, as it is in the United States.

**Table:** 

Mexican Americans are one of the fattest groups in what is one of the fattest nations on earth. Three out of four Mexican American adults (aged >20 years) were either overweight or obese at the end of the 20^th^ century (7). Plentiful and unhealthy diets, many hours of television watching, and a reluctance to exercise are some of the factors blamed. For example, a study of Mexican children along the Mexico–U.S. border showed low intake of fruits and vegetables and excessive consumption of soft drinks and high-fat snacks (8).

Obesity is an easy, visible marker of the worldwide pandemic of noncommunicable diseases for which considerable data from around the world are available (2). Obesity is also a major risk factor for type 2 diabetes, and where obesity is rising we can expect diabetes to follow (9).

## The Type 2 Diabetes Pandemic

Diabetes is a growing problem worldwide. The prevalence of diabetes in adults (aged >20 years) is projected to increase in developed countries from 6.0% in 1995 to 7.6% by 2025 (10). Diabetes in developing countries will also increase from 3.3% to 4.9%, and because of initial population sizes and growth, the increase in the number of people with diabetes will come disproportionately from the developing world. The number of individuals with diabetes will rise from 51 million to 72 million in developed countries, but the number will rise from 84 million to 228 million in developing countries. The three nations with the greatest numbers of individuals with diabetes in 1995 were India (19.4 million), China (16.0 million), and the United States (13.9 million). In 2025, the rankings will be unchanged, but the absolute number will increase dramatically in India (to 57.2 million) and China (to 37.6 million) and less so in the United States (to 21.9 million). Mexico, which was ninth in the world in 1995 (3.8 million), will rise to seventh place by 2025 (11.7 million).

**Figure 3:**
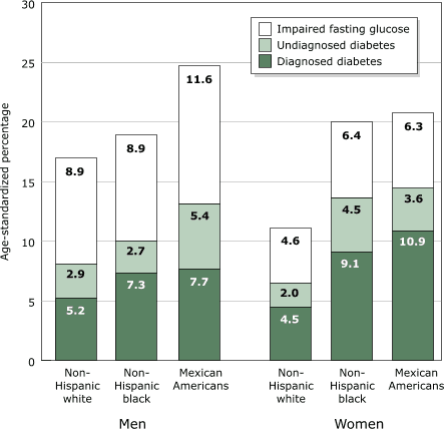
Age-standardized prevalence of diagnosed and undiagnosed diabetes and impaired fasting glucose in the U.S. population aged ≥20 years by sex and ethnic group, based on the Third National Health and Nutrition Examination Survey (NHANES III). Data from Harris et al, 1998 (12).

Diabetes is a serious public health problem among Mexicans and Mexican Americans. Diabetes was found in 8.1% of Mexican adults in 2000 (11) compared with 13.1% and 14.5% of Mexican American men and women in 1988–94 (12). In the United States, adults of Mexican origin, particularly men, had higher rates of prevalence of diabetes than non-Hispanic whites or blacks, as well as a greater degree of impaired fasting glucose (Figure 3). The prevalence of diabetes in the United States is rising rapidly. The prevalence of diabetes increased from 8.9% in 1976–1980 to 12.3% in 1988–94 among adults aged 40 to 74 (12). Mexican Americans, the largest Hispanic/Latino subgroup in the United States, are more than twice as likely to have diabetes as non-Hispanic whites of similar age (13).

**Table:** 

Born in Central America, I share a similar ancestry with Mexicans (Spanish and Amerindian). Not surprisingly, diabetes runs in my family. Some statistics should scare me. The lifetime risk of developing diabetes for U.S. individuals born in 2002 is about one in three for the general population, but about one in two for the Hispanic population (14).

## Ancestry and Prenatal Exposure

Lifestyle characteristics are primarily responsible for the high levels of obesity and diabetes among Mexicans, but other considerations are also important. The San Antonio Heart Study began in 1979 and is a population-based study of diabetes and cardiovascular disease in Mexican Americans and non-Hispanic whites in San Antonio, Texas (9). One of the interesting findings of the study is that the degree of Native American ancestry is a major risk factor for diabetes, presumably because of inherited thrifty genes (15).

**Figure 4:**
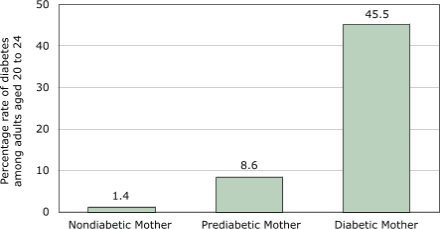
Prevalence of type 2 diabetes among Pima Indian adults, Arizona, aged 20 to 24, by diabetes status of the mother during pregnancy. A prediabetic mother is one who develops diabetes after delivery. Data from Pettitt et al, 1988 (16).

The role of intergenerational mechanisms, specifically the risk of developing diabetes in adulthood as a result of prenatal exposure to diabetes, has become clear from studies of Pima Indians in Arizona (Figure 4). The prevalence of diabetes among adults aged 20 to 24 was found to be 1.4% if the mother was free of diabetes, 8.6% if she was prediabetic (developed diabetes after delivery), and 45.5% if she had gestational diabetes (16). Follow-up studies over three decades reveal a steady rise in diabetes in Pima children and adolescents. From 1967–76 to 1987–96, the prevalence of diabetes in girls aged 10 to 14 years increased from 0.72% to 2.88%. In girls aged 15 to 19 years, the prevalence increased from 2.73% to 5.31% during the same period (17). The percentage of youths (aged 10 to 19 years) who were exposed to gestational diabetes increased during this period (Figure 5). In 1967–76, 2.1% of youths were exposed to gestational diabetes; by 1987–96, exposure had almost quadrupled to 7.5% of pregnancies. The fraction of diabetes attributable to gestational diabetes also rose markedly in youths aged 10 to 19 so that by 1987–96, more than one third of cases of diabetes could be attributed to gestational diabetes. Also, more than 70% of persons with prenatal exposure developed type 2 diabetes at 25 to 34 years of age (18). Clearly, the hyperglycemic intrauterine environment brought on by gestational diabetes is an important determinant of early-onset type 2 diabetes that is above any genetically transmitted susceptibility and is another example of fetal programming (19). An additional consequence is that 50% of women with gestational diabetes will themselves develop diabetes within five years (20). The concern about gestational diabetes is not limited to the Pima population. The incidence of gestational diabetes increased from 4.9% in 1990 to 7.1% in 2000 in California, where Asian and Hispanic women had higher incidences than whites and African Americans (20).

**Table:** 

**Figure 5:** Exposure to gestational diabetes (GD) and fraction of diabetes attributed to GD among cohorts of Pima Indian adults, Arizona, aged 10 to 19 years (n = 6902). Data from Dabelea et al, 1998 (17).

**Table:** 

	**1967–76**	**1977–86**	**1987–96**
**Exposure to GD**	2.1	4.0	7.5
**Attributable fraction**	18.1	23.7	35.4

Gestational diabetes is adding fuel to an already raging epidemic of diabetes. The intergenerational component operates through women and begins with the interaction of genetic susceptibility and unhealthy lifestyle practices that precipitate obesity in girls and women of reproductive age, which in turn increases the risk of diabetes prior to or during pregnancy. The percentage of women exposed to diabetes in their intrauterine life then increases in each subsequent generation, driving rates of diabetes in the general population higher and higher with each generation. This scenario is already unfolding in the Mexican populations of North America and deserves serious study.

## Where Do We Go From Here?

The costs of diabetes in the United States were estimated at $132 billion for 2002 (21). Meeting the demand for public health care services caused by diabetes will alone cost Mexico $318 million in 2005, 26% more than in 2003 (22). While the monetary costs are staggering, the suffering and disability among those afflicted with diabetes and their families are incalculable.

We need to confront the diabetes pandemic with urgency. Efficacy studies show that lifestyle changes can effectively reduce the incidence of diabetes in persons at high risk (23). We need effective programs that promote healthy lifestyles and make screening and sound case management widely available. We also need to devote significant resources to developing new drugs and therapies. Combating obesity and inactivity must become a national priority. Preventive actions must be undertaken along a broad front, impacting behavior as well as the physical environment — from how we design our cities to promote physical activity to what agriculture and food policies we support to foster a healthier food basket. We need to promote aggressively a love of physical activity and healthy diets, particularly among our children. We need flexible programs that can fit local settings and our diversity of cultures, including the mosaic of Hispanic groups in the United States. Mexico, with far fewer resources, must do all of the above while combating yesterday's unresolved problems of undernutrition. The future will be grim only if we let it become so.
